# The genome sequence of a solitary sea squirt, *Ascidia mentula *(Müller, 1776)

**DOI:** 10.12688/wellcomeopenres.20415.1

**Published:** 2023-12-29

**Authors:** John Bishop, Christine Wood, Marine Biological Association Genome Acquisition Lab, Darwin Tree of Life Barcoding collective, Wellcome Sanger Institute Tree of Life Management, Samples and Laboratory team, Wellcome Sanger Institute Scientific Operations: Sequencing Operations, Wellcome Sanger Institute Tree of Life Core Informatics team, Tree of Life Core Informatics collective, Darwin Tree of Life Consortium

**Affiliations:** 1 The Marine Biological Association, Plymouth, England, UK

**Keywords:** Ascidia mentula, (a solitary sea squirt), genome sequence, chromosomal, Phlebobranchia

## Abstract

We present a genome assembly from an individual *Ascidia mentula* (the (a solitary sea squirt); Chordata; Ascidiacea; Phlebobranchia; Ascidiidae). The genome sequence is 197.0 megabases in span. Most of the assembly is scaffolded into 9 chromosomal pseudomolecules. The mitochondrial genome has also been assembled and is 19.46 kilobases in length.

## Species taxonomy

Eukaryota; Metazoa; Eumetazoa; Bilateria; Deuterostomia; Chordata; Tunicata; Ascidiacea; Phlebobranchia; Ascidiidae; *Ascidia*; *Ascidia mentula* (Müller, 1776) (NCBI:txid38561).

## Background

*Ascidia mentula* is a unitary (= ‘solitary’, non-budding) ascidian (sea squirt) of the order Phlebobranchia. The body is oblong or elongate-ovoid and grows to a size of about 180 mm. The external covering, the tunic, is thick and translucent, with irregular swellings. The openings of the two siphons are very well separated, the inhalant siphon being at the extreme end of the body and the exhalant one-half to two-thirds of the way back; the siphon openings are barely raised above the general outline of the tunic. A network of fine vascular sinuses, with multiple branches each ending in a minute bulb near the surface, imparts pink or red colouration to the tunic. This colouration appears to be influenced by the amount of light experienced by the individual, ranging from red when well-lit through pink to grey (but still with tiny pink/red spots) in dark sites. As is typical of unitary ascidians, eggs and sperm are spawned for external fertilisation.

*A. mentula* occurs from Norway to the Mediterranean and Black Seas, and from the lower shore to a depth of around 200 m, attached to solid objects (rock, stones, shells, etc.). Aspects of anatomy and development of the species’ larva were the subject of detailed studies in the late 19th century ( Kowalevsky, 1871; Kupffer, 1872; Willey, 1893). Subsequent studies have addressed larval behaviour (e.g. Svane *et al.*, 1987), fertilisation biology (e.g. Fukumoto & Zarnescu, 2003; Havenhand, 1991), feeding (e.g. Fiala-Medioni, 1978) population dynamics (e.g. ( Svane, 1984), the accumulation of vanadium in the haemolymph (e.g. Pirie & Bell, 1984) and natural products (e.g. Aiello *et al.*, 1997).

## Genome sequence report

**Figure 1.**
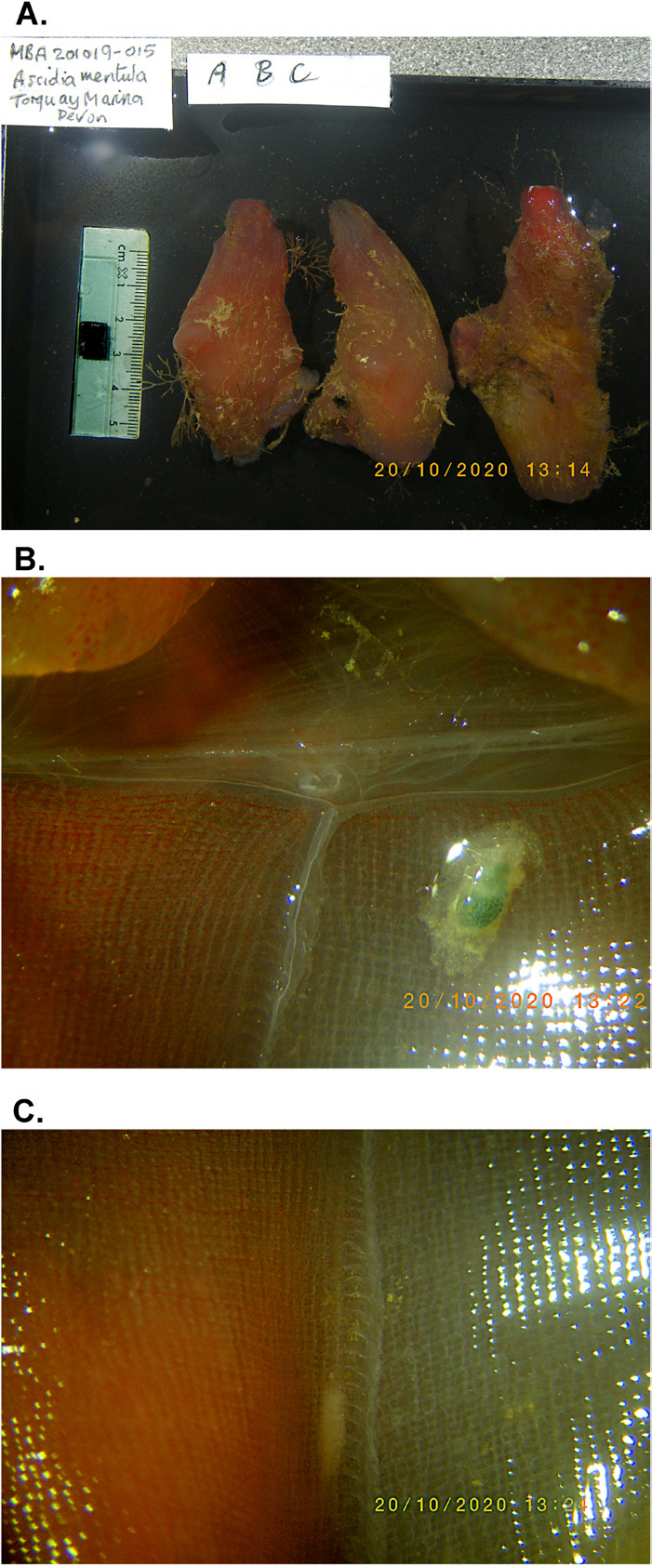
Photographs of the *Ascidia mentula* (kaAscMent1) specimen used for genome sequencing. **A**: Three specimens of *Ascidia mentula* collected at the same time from Torquay Marina. The specimen sequenced, 'A', is on the left. **B**: Dissection showing anterior portion of branchial basket with dorsal lamina, circumpharyngeal band, dorsal tubercle and parasitic copepod (green). **C**: Dissection showing portion of branchial basket posterior to that shown in B, with mucus string (food string) adjacent to dorsal lamina.

The genome was sequenced from one *Ascidia mentula* ( Figure 1) collected from Torquay (50.46, –3.53). A total of 74-fold coverage in Pacific Biosciences single-molecule HiFi long reads was generated. Primary assembly contigs were scaffolded with chromosome conformation Hi-C data. Manual assembly curation corrected 27 missing joins or mis-joins, reducing the scaffold number by 12.73%, and decreasing the scaffold N50 by 7.49%.

**Figure 2.**
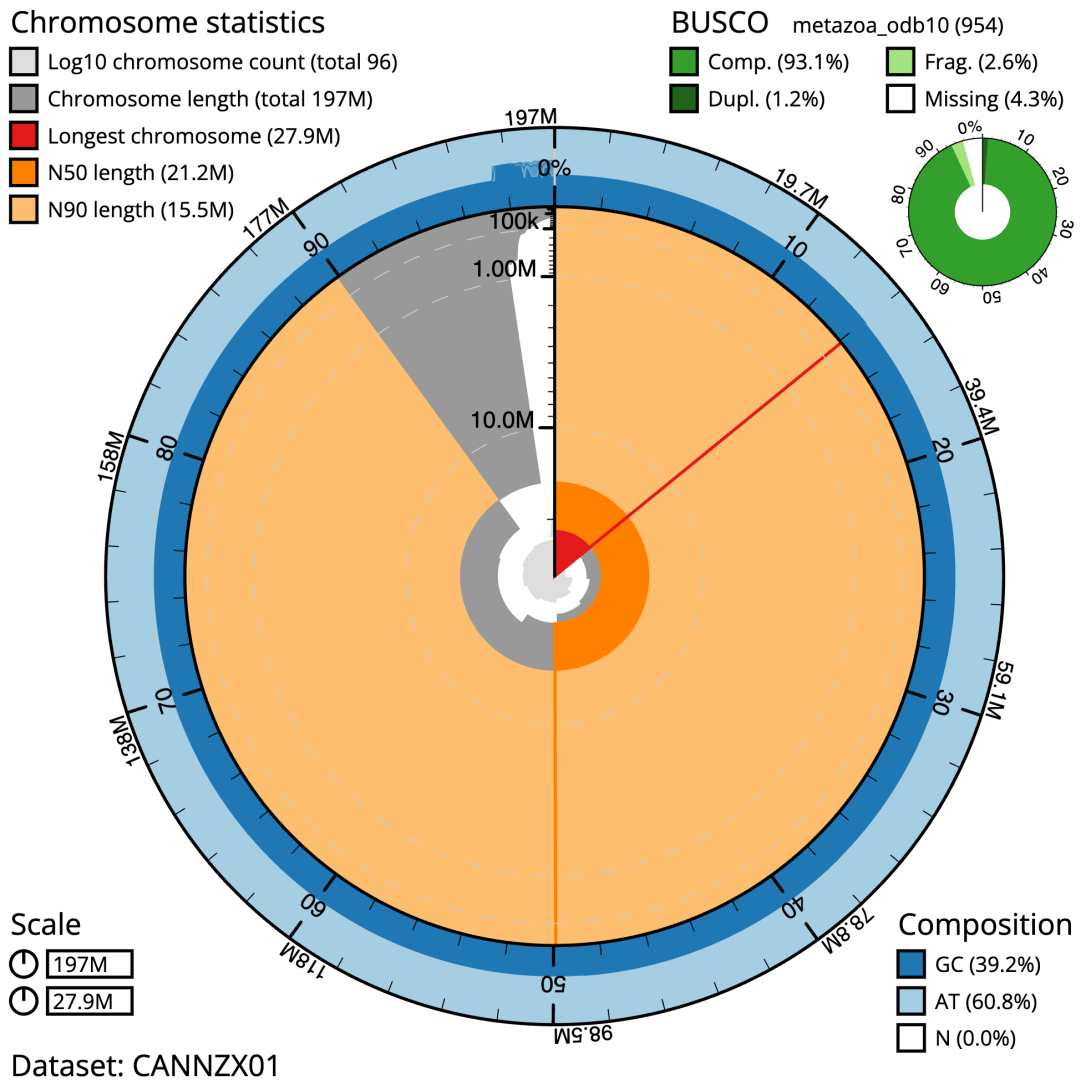
Genome assembly of *Ascidia mentula*, kaAscMent1.1: metrics. The BlobToolKit Snailplot shows N50 metrics and BUSCO gene completeness. The main plot is divided into 1,000 size-ordered bins around the circumference with each bin representing 0.1% of the 196,975,169 bp assembly. The distribution of scaffold lengths is shown in dark grey with the plot radius scaled to the longest scaffold present in the assembly (27,920,005 bp, shown in red). Orange and pale-orange arcs show the N50 and N90 scaffold lengths (21,171,019 and 15,452,589 bp), respectively. The pale grey spiral shows the cumulative scaffold count on a log scale with white scale lines showing successive orders of magnitude. The blue and pale-blue area around the outside of the plot shows the distribution of GC, AT and N percentages in the same bins as the inner plot. A summary of complete, fragmented, duplicated and missing BUSCO genes in the metazoa_odb10 set is shown in the top right. An interactive version of this figure is available at https://blobtoolkit.genomehubs.org/view/Ascidia%20mentula/dataset/CANNZX01/snail.

**Figure 3.**
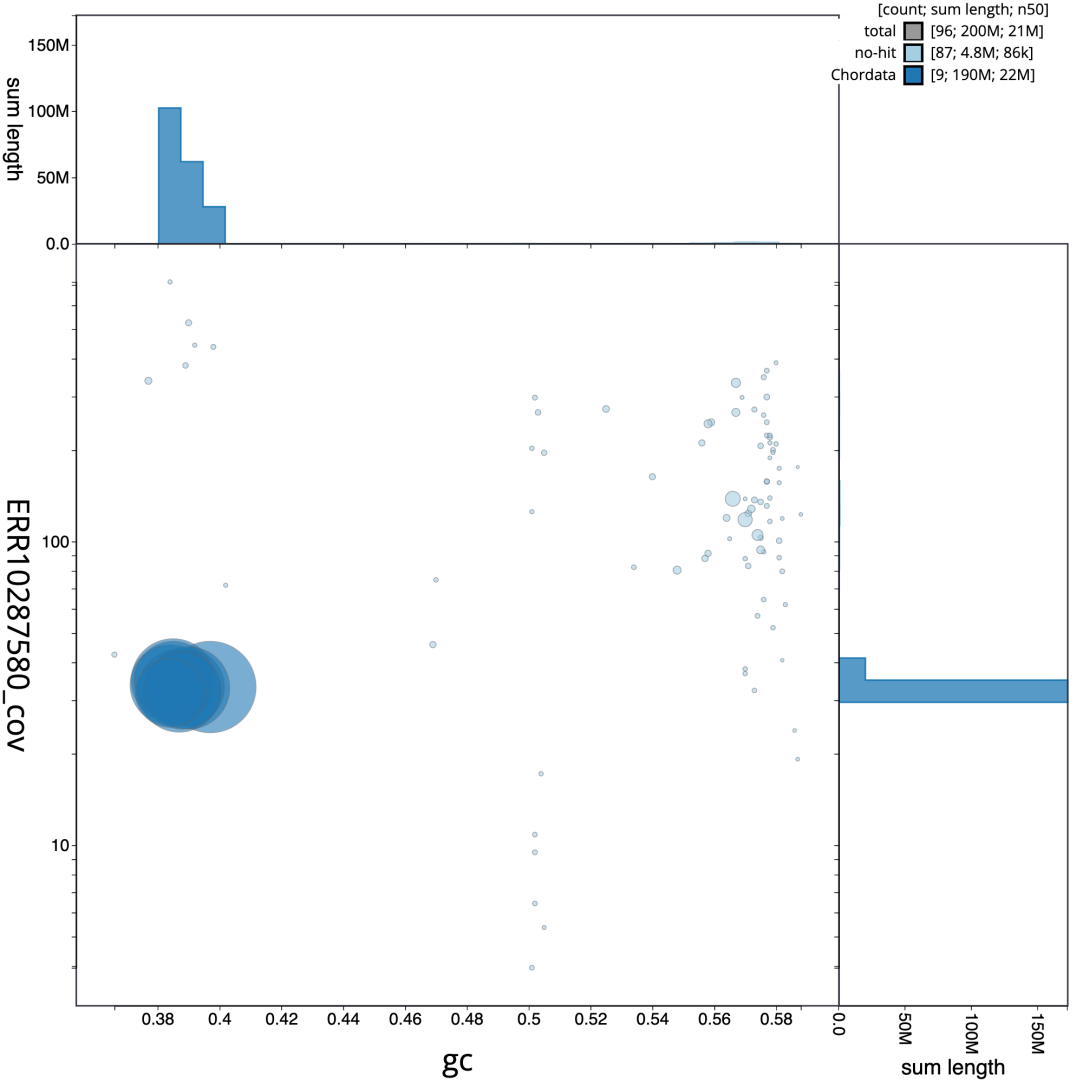
Genome assembly of *Ascidia mentula*, kaAscMent1.1: BlobToolKit GC-coverage plot. Scaffolds are coloured by phylum. Circles are sized in proportion to scaffold length. Histograms show the distribution of scaffold length sum along each axis. An interactive version of this figure is available at https://blobtoolkit.genomehubs.org/view/Ascidia%20mentula/dataset/CANNZX01/blob.

**Figure 4.**
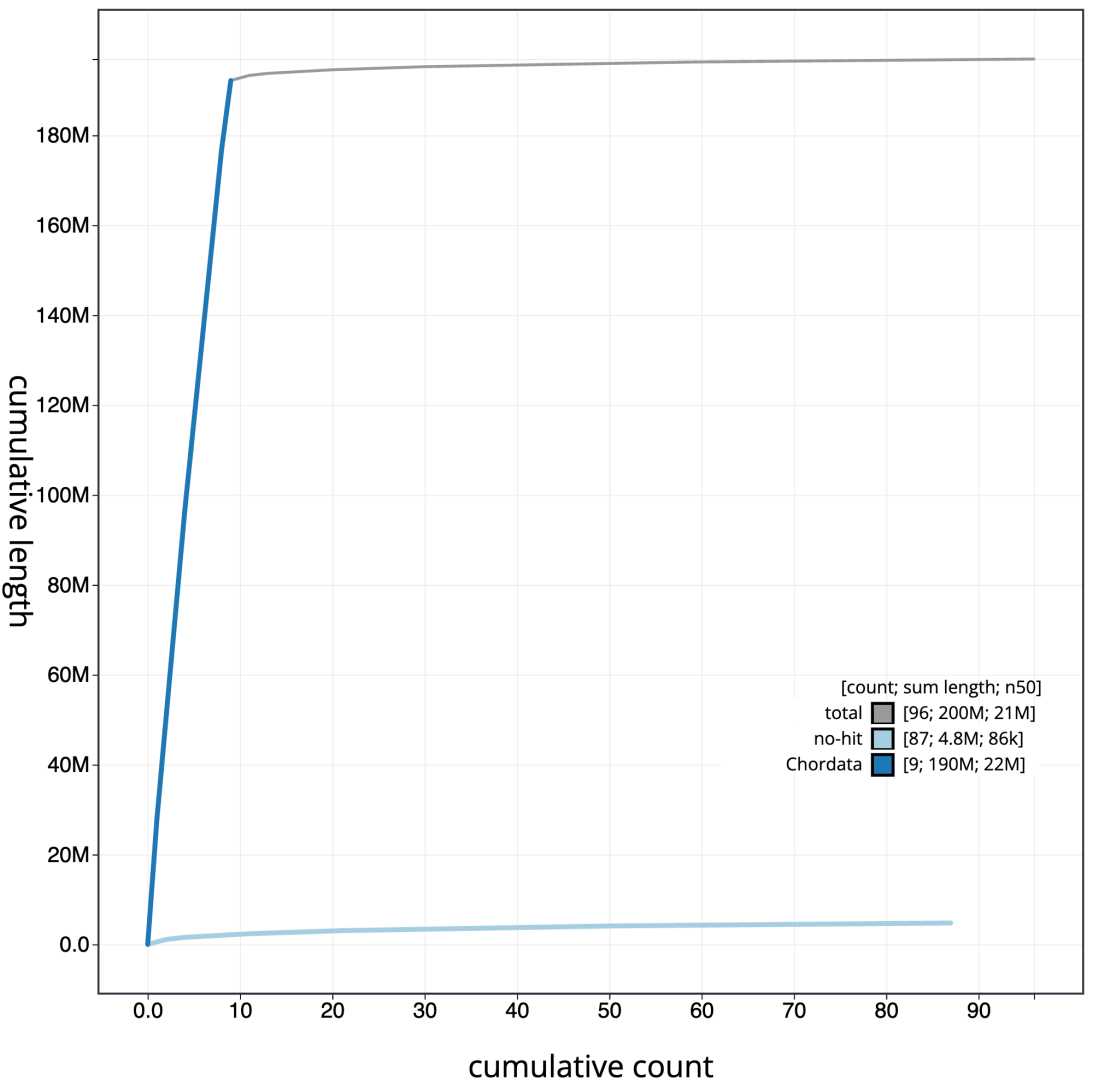
Genome assembly of *Ascidia mentula*, kaAscMent1.1: BlobToolKit cumulative sequence plot. The grey line shows cumulative length for all scaffolds. Coloured lines show cumulative lengths of scaffolds assigned to each phylum using the buscogenes taxrule. An interactive version of this figure is available at https://blobtoolkit.genomehubs.org/view/Ascidia%20mentula/dataset/CANNZX01/cumulative.

**Figure 5.**
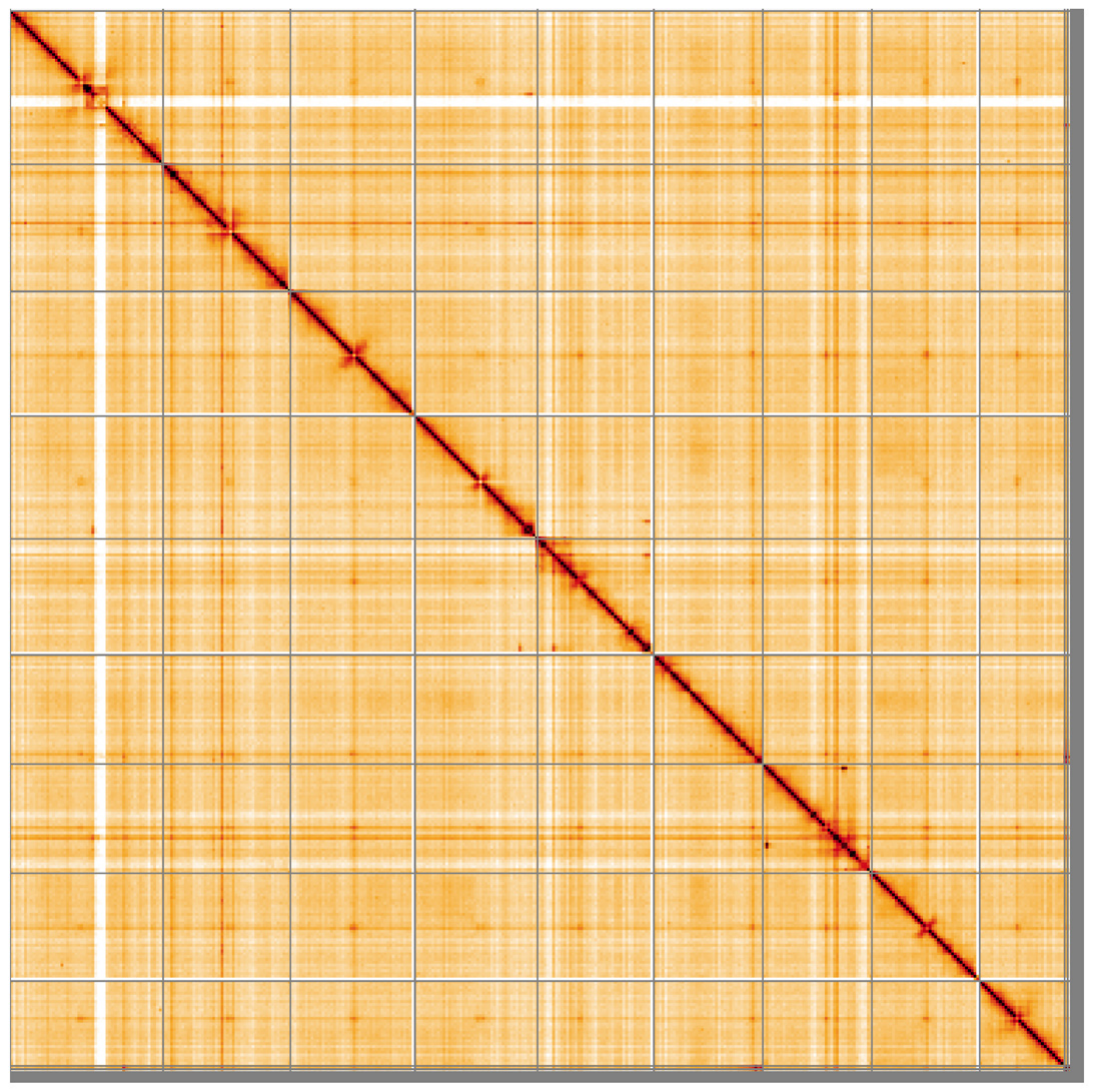
Genome assembly of *Ascidia mentula*, kaAscMent1.1: Hi-C contact map of the kaAscMent1.1 assembly, visualised using HiGlass. Chromosomes are shown in order of size from left to right and top to bottom. An interactive version of this figure may be viewed at https://genome-note-higlass.tol.sanger.ac.uk/l/?d=Io-Iy42sQcqV0NQQVaocWQ.

The final assembly has a total length of 197.0 Mb in 95 sequence scaffolds with a scaffold N50 of 21.2 Mb ( Table 1). A summary of the assembly statistics is shown in Figure 2, while the distribution of assembly scaffolds on GC proportion and coverage is shown in Figure 3. The cumulative assembly plot in Figure 4 shows curves for subsets of scaffolds assigned to different phyla. Most (99.9%) of the assembly sequence was assigned to 9 chromosomal-level scaffolds. Chromosome-scale scaffolds confirmed by the Hi-C data are named in order of size ( Figure 5; Table 2). While not fully phased, the assembly deposited is of one haplotype. Contigs corresponding to the second haplotype have also been deposited. The mitochondrial genome was also assembled and can be found as a contig within the multifasta file of the genome submission.

**Table 1.** Genome data for *Ascidia mentula*, kaAscMent1.1.

Project accession data		
Assembly identifier	kaAscMent1.1	
Assembly release date	2022-12-11	
Species	*Ascidia mentula*	
Specimen	kaAscMent1	
NCBI taxonomy ID	38561	
BioProject	PRJEB56132	
BioSample ID	SAMEA8724667	
Isolate information	kaAscMent1: other somatic tissue (DNA sequencing, Hi-C scaffolding and RNA sequencing)	
Assembly metrics *		*Benchmark*
Consensus quality (QV)	64.9	*≥ 50*
*k*-mer completeness	100%	*≥ 95%*
BUSCO **	C:93.1%[S:91.9%,D:1.2%], F:2.6%,M:4.3%,n:954	*C ≥ 95%*
Percentage of assembly mapped to chromosomes	99.9%	*≥ 95%*
Sex chromosomes	-	*localised homologous pairs*
Organelles	Mitochondrial genome assembled	*complete single alleles*
Raw data accessions		
PacificBiosciences SEQUEL II	ERR10287580	
Hi-C Illumina	ERR10297860	
PolyA RNA-Seq Illumina	ERR10378036	
Genome assembly		
Assembly accession	GCA_947561715.1	
*Accession of alternate haplotype*	GCA_947561685.1	
Span (Mb)	197.0	
Number of contigs	128	
Contig N50 length (Mb)	11.9	
Number of scaffolds	95	
Scaffold N50 length (Mb)	21.2	
Longest scaffold (Mb)	27.9	

* Assembly metric benchmarks are adapted from column VGP-2020 of “Table 1: Proposed standards and metrics for defining genome assembly quality” from ( Rhie *et al.*, 2021). ** BUSCO scores based on the metazoa_odb10 BUSCO set using v5.3.2. C = complete [S = single copy, D = duplicated], F = fragmented, M = missing, n = number of orthologues in comparison. A full set of BUSCO scores is available at https://blobtoolkit.genomehubs.org/view/Ascidia%20mentula/dataset/CANNZX01/busco.

**Table 2.** Chromosomal pseudomolecules in the genome assembly of *Ascidia mentul* *a*, kaAscMent1.

INSDC accession	Chromosome	Length (Mb)	GC%
OX387197.1	1	27.92	39.5
OX387198.1	2	19.88	39.0
OX387199.1	3	23.17	38.5
OX387200.1	4	22.72	38.5
OX387201.1	5	22.35	39.0
OX387202.1	6	21.17	38.5
OX387203.1	7	19.87	38.5
OX387204.1	8	19.66	39.0
OX387205.1	9	15.45	38.5
OX387206.1	MT	0.02	38.5

The estimated Quality Value (QV) of the final assembly is 64.9 with *k*-mer completeness of 100%, and the assembly has a BUSCO v5.3.2 completeness of 93.1% (single = 91.9%, duplicated = 1.2%), using the metazoa_odb10 reference set ( *n* = 954).

Metadata for specimens, spectral estimates, sequencing runs, contaminants and pre-curation assembly statistics can be found at https://links.tol.sanger.ac.uk/species/38561.

## Methods

### Sample acquisition and nucleic acid extraction

An *Ascidia mentula* (specimen ID MBA-201019-015A, ToLID kaAscMent1) was collected by hand from Torquay Marina, Devon, UK (latitude 50.46, longitude –3.53) on 2020-10-19. The specimen was taken from the marina pontoon (on submerged plastic settlement panels) by Christine Wood and John Bishop (Marine Biological Association). The specimen was identified by John Bishop and preserved in liquid nitrogen.

The workflow for high molecular weight (HMW) DNA extraction at the Wellcome Sanger Institute (WSI) includes a sequence of core procedures: sample preparation; sample homogenisation; DNA extraction; HMW DNA fragmentation; and fragmented DNA clean-up. The kaAscMent1 sample was weighed and dissected on dry ice with tissue set aside for Hi-C sequencing (as per the protocol at https://dx.doi.org/10.17504/protocols.io.x54v9prmqg3e/v1). For sample homogenisation, somatic tissue was cryogenically disrupted using the Sample Homogenisation: Covaris cryoPREP® Automated Dry Pulverizer protocol ( https://dx.doi.org/10.17504/protocols.io.eq2lyjp5qlx9/v1). HMW DNA was extracted by means of the Automated MagAttract protocol ( https://dx.doi.org/10.17504/protocols.io.kxygx3y4dg8j/v1). HMW DNA was sheared into an average fragment size of 12–20 kb in a Megaruptor 3 system with speed setting 30, following the HMW DNA Fragmentation: Diagenode Megaruptor®3 for PacBio HiFi protocol ( https://dx.doi.org/10.17504/protocols.io.8epv5x2zjg1b/v1). Sheared DNA was purified following either the Manual solid-phase reversible immobilisation (SPRI) protocol ( https://dx.doi.org/10.17504/protocols.io.kxygx3y1dg8j/v1), or the Automated SPRI protocol ( https://dx.doi.org/10.17504/protocols.io.q26g7p1wkgwz/v1) for higher throughput. In brief, the method employs a 1.8X ratio of AMPure PB beads to sample to eliminate shorter fragments and concentrate the DNA. The concentration of the sheared and purified DNA was assessed using a Nanodrop spectrophotometer and Qubit Fluorometer and Qubit dsDNA High Sensitivity Assay kit. Fragment size distribution was evaluated by running the sample on the FemtoPulse system.

RNA was extracted from tissue of kaAscMent in the Tree of Life Laboratory at the WSI using the RNA Extraction: Automated MagMax™ *mir*Vana protocol ( https://dx.doi.org/10.17504/protocols.io.6qpvr36n3vmk/v1). The RNA concentration was assessed using a Nanodrop spectrophotometer and Qubit Fluorometer using the Qubit RNA Broad-Range (BR) Assay kit. Analysis of the integrity of the RNA was done using the Agilent RNA 6000 Pico Kit and Eukaryotic Total RNA assay.

Protocols employed by the Tree of Life laboratory are publicly available on protocols.io: https://dx.doi.org/10.17504/protocols.io.8epv5xxy6g1b/v1.

### Sequencing

Pacific Biosciences HiFi circular consensus DNA sequencing libraries were constructed according to the manufacturers’ instructions. Poly(A) RNA-Seq libraries were constructed using the NEB Ultra II RNA Library Prep kit. DNA and RNA sequencing was performed by the Scientific Operations core at the WSI on Pacific Biosciences SEQUEL II (HiFi) and Illumina NovaSeq 6000 (RNA-Seq) instruments. Hi-C data were also generated from somatic tissue of kaAscMent1 using the Arima2 kit and sequenced on the Illumina NovaSeq 6000 instrument.

### Genome assembly, curation and evaluation

Assembly was carried out with Hifiasm ( Cheng *et al.*, 2021) and haplotypic duplication was identified and removed with purge_dups ( Guan *et al.*, 2020). The assembly was then scaffolded with Hi-C data ( Rao *et al.*, 2014) using YaHS ( Zhou *et al.*, 2023). The assembly was checked for contamination and corrected as described previously ( Howe *et al.*, 2021). Manual curation was performed using HiGlass ( Kerpedjiev *et al.*, 2018) and Pretext ( Harry, 2022). The mitochondrial genome was assembled using MitoHiFi ( Uliano-Silva *et al.*, 2023), which runs MitoFinder ( Allio *et al.*, 2020) or MITOS ( Bernt *et al.*, 2013) and uses these annotations to select the final mitochondrial contig and to ensure the general quality of the sequence.

A Hi-C map for the final assembly was produced using bwa-mem2 ( Vasimuddin *et al.*, 2019) in the Cooler file format ( Abdennur & Mirny, 2020). To assess the assembly metrics, the *k*-mer completeness and QV consensus quality values were calculated in Merqury ( Rhie *et al.*, 2020). This work was done using Nextflow ( Di Tommaso *et al.*, 2017) DSL2 pipelines “sanger-tol/readmapping” ( Surana *et al.*, 2023a) and “sanger-tol/genomenote” ( Surana *et al.*, 2023b). The genome was analysed within the BlobToolKit environment ( Challis *et al.*, 2020) and BUSCO scores ( Manni *et al.*, 2021; Simão *et al.*, 2015) were calculated.

Table 3 contains a list of relevant software tool versions and sources.

**Table 3.** Software tools: versions and sources.

Software tool	Version	Source
BlobToolKit	4.1.7	https://github.com/blobtoolkit/blobtoolkit
BUSCO	5.3.2	https://gitlab.com/ezlab/busco
Hifiasm	0.16.1-r375	https://github.com/chhylp123/hifiasm
HiGlass	1.11.6	https://github.com/higlass/higlass
Merqury	MerquryFK	https://github.com/thegenemyers/MERQURY.FK
MitoHiFi	2	https://github.com/marcelauliano/MitoHiFi
PretextView	0.2	https://github.com/wtsi-hpag/PretextView
purge_dups	1.2.3	https://github.com/dfguan/purge_dups
sanger-tol/genomenote	v1.0	https://github.com/sanger-tol/genomenote
sanger-tol/readmapping	1.1.0	https://github.com/sanger-tol/readmapping/tree/1.1.0
YaHS	1.1a.2	https://github.com/c-zhou/yahs

### Wellcome Sanger Institute – Legal and Governance

The materials that have contributed to this genome note have been supplied by a Darwin Tree of Life Partner. The submission of materials by a Darwin Tree of Life Partner is subject to the **‘Darwin Tree of Life Project Sampling Code of Practice’**, which can be found in full on the Darwin Tree of Life website here. By agreeing with and signing up to the Sampling Code of Practice, the Darwin Tree of Life Partner agrees they will meet the legal and ethical requirements and standards set out within this document in respect of all samples acquired for, and supplied to, the Darwin Tree of Life Project.

Further, the Wellcome Sanger Institute employs a process whereby due diligence is carried out proportionate to the nature of the materials themselves, and the circumstances under which they have been/are to be collected and provided for use. The purpose of this is to address and mitigate any potential legal and/or ethical implications of receipt and use of the materials as part of the research project, and to ensure that in doing so we align with best practice wherever possible. The overarching areas of consideration are:

- Ethical review of provenance and sourcing of the material
- Legality of collection, transfer and use (national and international)

Each transfer of samples is further undertaken according to a Research Collaboration Agreement or Material Transfer Agreement entered into by the Darwin Tree of Life Partner, Genome Research Limited (operating as the Wellcome Sanger Institute), and in some circumstances other Darwin Tree of Life collaborators.

## Data Availability

European Nucleotide Archive: *Ascidia mentula* (a solitary sea squirt). Accession number PRJEB56132; https://identifiers.org/ena.embl/PRJEB56132 ( Wellcome Sanger Institute, 2022). The genome sequence is released openly for reuse. The *Ascidia mentula* genome sequencing initiative is part of the Darwin Tree of Life (DToL) project. All raw sequence data and the assembly have been deposited in INSDC databases. The genome will be annotated using available RNA-Seq data and presented through the Ensembl pipeline at the European Bioinformatics Institute. Raw data and assembly accession identifiers are reported in Table 1.

## Author information

Members of the Marine Biological Association Genome Acquisition Lab are listed here: https://doi.org/10.5281/zenodo.8382513.

Members of the Darwin Tree of Life Barcoding collective are listed here: https://doi.org/10.5281/zenodo.4893703.

Members of the Wellcome Sanger Institute Tree of Life Management, Samples and Laboratory Team are listed here: https://doi.org/10.5281/zenodo.10066175.

Members of Wellcome Sanger Institute Scientific Operations: Sequencing Operations are listed here: https://doi.org/10.5281/zenodo.10043364.

Members of the Wellcome Sanger Institute Tree of Life Core Informatics team are listed here: https://doi.org/10.5281/zenodo.10066637.

Members of the Tree of Life Core Informatics collective are listed here: https://doi.org/10.5281/zenodo.5013541.

Members of the Darwin Tree of Life Consortium are listed here: https://doi.org/10.5281/zenodo.4783558.
