# Supplementary material for: The genome sequence of a solitary sea squirt, Ascidia mentula (Müller, 1776)
Source: Wellcome Open Res. 2023 Dec 29;8:583. [Version 1] doi: 10.12688/wellcomeopenres.20415.1 (PMC11109543; doi:10.12688/wellcomeopenres.20415.1)
Supplement: Peer review history

# Reviewer report: Reviewer response for version 1

**DOI:** 10.21956/wellcomeopenres.22604.r74729

**Published:** 2024-05-11

**Authors:** Angelica Crottini

**Affiliations:** 1 Universidade do Porto, Porto, Porto District, Portugal

This note reports on the genome assembly of *Ascidia mentula*, a solitary ascidian belonging to the order Phlebobranchia. The genome of *Ascidia mentula* is assembled in 9 chromosomes and all steps, from collecting to genome sequencing and assembly, are reported in a clear and straightforward way.

 Here below are a few minor suggestions:

 “(50.46, –3.53)” change to “(latitude 50.46, longitude –3.53)”

 Table 2 caption: add “and mitochondrial genome”

 References

 - Svane IB remove uppercase?

 - Willey A: Ascidia mentula should be in italics

Are sufficient details of methods and materials provided to allow replication by others?

Yes

Is the rationale for creating the dataset(s) clearly described?

Yes

Are the datasets clearly presented in a useable and accessible format?

Yes

Are the protocols appropriate and is the work technically sound?

Yes

Reviewer Expertise:

Taxonomy, Systematics, Evolutionary Genetics/Genomics

I confirm that I have read this submission and believe that I have an appropriate level of expertise to confirm that it is of an acceptable scientific standard.

# Reviewer report: Reviewer response for version 1

**DOI:** 10.21956/wellcomeopenres.22604.r72964

**Published:** 2024-02-21

**Authors:** Alan Le Moan

**Affiliations:** 1 UMR7144, Sorbonne Universite, Roscoff, Bretagne, France; 2 Department of Marine Sciences, Tjärnö Marine Laboratory, Gothenburg University, 452 96 Strömstad, Strömstad,, Sweden

This note presents the genome assembly of the solitary tunicate *Ascidia mentula*. The genome is of high quality, assembled into 9 chromosomes representing 99.9% of the total assembly, and will be useful for the community interested in comparative genomics and tunicate.

 In what follows, I am listing a few minor elements:

 Background:

 “the amount of light”: is it artificial or sun light? I am not sure is very relevant, but as tunicates are very common in artificial habitat, this information could be added.  I guess a reference could  be included too.

 “Subsequent studies have addressed … natural products” I am not sure that I see what this meant here

 Method:

 In the sentence: “In brief, the method employs a 1.8X ratio of AMPure PB beads to sample to eliminate shorter fragments and concentrate the DNA. “  to sample or to eliminate -> choose one not both?  Probably to eliminate

 “Protocols employed by the Tree of Life laboratory are publicly available on protocols.io: https://dx.doi.org/10.17504/protocols.io.8epv5xxy6g1b/v1.”  I am not very sure this is needed here as they are cited throughout the text?

 “The mitochondrial genome was assembled using MitoHiFi ( Uliano-Silva *et al.*, 2023), which runs MitoFinder ( Allio *et al.*, 2020) or MITOS ( Bernt *et al.*, 2013) and uses these annotations to select the final mitochondrial contig and to ensure the general quality of the sequence.” I am not sure this is clear enough here. In the end, did you use both Mitofinder and MITOS? And I am not sure what is the purpose of having both.

Are sufficient details of methods and materials provided to allow replication by others?

Yes

Is the rationale for creating the dataset(s) clearly described?

Yes

Are the datasets clearly presented in a useable and accessible format?

Yes

Are the protocols appropriate and is the work technically sound?

Yes

Reviewer Expertise:

Population genomics

I confirm that I have read this submission and believe that I have an appropriate level of expertise to confirm that it is of an acceptable scientific standard.
